# Interdisciplinary perspectives on the development, integration, and application of cognitive ontologies

**DOI:** 10.3389/fninf.2014.00062

**Published:** 2014-06-20

**Authors:** Janna Hastings, Gwen A. Frishkoff, Barry Smith, Mark Jensen, Russell A. Poldrack, Jane Lomax, Anita Bandrowski, Fahim Imam, Jessica A. Turner, Maryann E. Martone

**Affiliations:** ^1^European Molecular Biology Laboratory – European Bioinformatics Institute, Wellcome Trust Genome CampusHinxton, UK; ^2^Department of Philosophy and Swiss Center for Affective Sciences, University of GenevaSwitzerland; ^3^Evolutionary Bioinformatics, Swiss Institute for BioinformaticsLausanne, Switzerland; ^4^Department of Psychology/Neuroscience Institute, Georgia State UniversityGA, USA; ^5^Department of Philosophy and National Center for Ontological Research, University at BuffaloNY, USA; ^6^Imaging Research Center, University of Texas at AustinTX, USA; ^7^Department of Psychology, University of Texas at AustinTX, USA; ^8^Department of Neuroscience, University of Texas at AustinTX, USA; ^9^Neuroinformatics Framework Project, University of CaliforniaSan Diego, CA, USA; ^10^Mind Research NetworkAlbuquerque, NM, USA

**Keywords:** ontology, cognition, mental functioning, neuroscience, annotation, integration, big data, brain science

## Abstract

We discuss recent progress in the development of cognitive ontologies and summarize three challenges in the coordinated development and application of these resources. Challenge 1 is to adopt a standardized definition for cognitive processes. We describe three possibilities and recommend one that is consistent with the standard view in cognitive and biomedical sciences. Challenge 2 is harmonization. Gaps and conflicts in representation must be resolved so that these resources can be combined for mark-up and interpretation of multi-modal data. Finally, Challenge 3 is to test the utility of these resources for large-scale annotation of data, search and query, and knowledge discovery and integration. As term definitions are tested and revised, harmonization should enable coordinated updates across ontologies. However, the true test of these definitions will be in their community-wide adoption which will test whether they support valid inferences about psychological and neuroscientific data.

## INTRODUCTION

Mental processes unfold at multiple levels, from the cellular dynamics of memory consolidation to sensory and motor behaviors that engage the entire brain and body. Thus, a comprehensive understanding of the phenomena associated with cognition requires coordinated contributions from at least psychology, biology, and neuroscience (Albus et al., 2007). The last two decades have seen unprecedented steps toward this goal, with accelerated use of neuroimaging (PET and fMRI), neurophysiology (EEG), and neurogenomics, together with experimental paradigms from cognitive, clinical, and neuro-psychology (Jones and Mendell, 1999).

While these methods have produced a wealth of high-quality data about mental functioning^1^, major advances will require not just more data, but new ways of integrating, processing, and understanding the data (Decety and Cacioppo, 2010; Akil et al., 2011). Recently, ontologies have emerged as a key tool to support these efforts. An ontology is a formal representation of the types of entities in a given domain, along with their properties and interrelationships. Recent projects, such as the Cognitive Paradigm Ontology (CogPO, Turner and Laird, 2012), Neural Electromagnetic Ontologies (NEMO, Frishkoff et al., 2009, 2011a,b), the Neuroscience Information Framework (NIF, Gardner et al., 2008; Imam et al., 2012), and the Cognitive Atlas (Poldrack et al., 2011), show that ontologies can be used to link data across neuroscientific databases and other resources (e.g., Imam et al., 2012) and to facilitate meta-analyses of neuroimaging data (e.g., Laird et al., 2009; Frishkoff et al., 2011a; Fox and Friston, 2012; Turner and Laird, 2012).

The present paper summarizes these recent efforts and describes three challenges in the development and integration of cognitive ontologies. *Challenge 1* is to adopt standardized definitions for cognitive processes, and integrate cognitive processes within the framework offered by a foundation (upper-level) ontology shared with other biomedical ontologies. We describe three possibilities and recommend one that is consistent with the standard view in cognitive and biomedical sciences.

*Challenge 2* is harmonization of terms across ontologies. A search for “memory” in BioPortal (Noy et al., 2009) returns over 150 terms from 30 ontologies^2^. These and other basic terms, such as “perception,” “planning,” and “emotion,” have multiple and sometimes conflicting definitions across ontologies. These conflicts must be resolved so that multiple ontologies can be combined for mark-up and interpretation of multi-modal data. In the present paper, we describe the scope of several cognitive and biomedical ontologies and suggest areas for improved coordination in their development and application.

*Challenge 3* is to test the utility of cognitive ontologies for large-scale data annotation, search and query, and ontology-based analysis. While Challenge 1 is concerned with ontology development, Challenges 2 and 3 reflect an interest in ontology harmonization and application. As term definitions are tested and revised, harmonization should enable coordinated updates across ontologies. However, the true test of these definitions will be whether they support valid inferences about psychological and neuroscientific data. We conclude with our view on the future of the development and application of cognitive ontologies.

## CHALLENGE #1: DEFINING AND CLASSIFYING MENTAL PROCESSES

Understanding the relationship between mental functioning and brain activity is a unifying aim for biology, neuroscience, psychology, and related fields. Upper-level ontologies, such as the Basic Formal Ontology (BFO; Smith and Grenon, 2004), can provide domain-independent integrating classes and domain-bridging relationships that can serve as a starting point for the annotation of data arising from this difficult search for understanding. Anatomical entities such as the central nervous system are classified as objects within BFO, and BFO also allows for “fiat” object parts for the classification of entities that have been arbitrarily (but usefully) divided into sections, e.g., the nervous system is divided into the central and peripheral nervous systems, although they are physically continuous through the connections of neurons residing in each division. Brain activity is classified as a process in BFO, and the anatomical entities are related via a participation relation to those processes.

Within BFO there are three possible ways to represent the relationship between mental processes and brain processes (Frishkoff, 2012). According to the first view, mental processes are distinct from physical (e.g., brain) processes. This is a folk psychological view and represents a kind of dualism: there are physical entities, and there are nonphysical (mental) entities. In contrast, the second and third views represent mental processes as a subclass of physiological (bodily) processes. The second view characterizes mental processes as a type of bodily process that is distinct from brain processes, while the third view holds that mental processes are a subtype of brain processes.

The first view, dualism, may generate hypotheses that are difficult or impossible to test within a scientific framework and is justifiably scientifically unpopular. In contrast, the second and third views are consistent with what we know about the physiological basis of cognition. Both assert that mental processes are physical processes, while the third view goes one step further in asserting that mental processes unfold solely in the brain, not the autonomic (reproductive, digestive) system or peripheral sensory and motor areas (e.g., hands, feet). This view is consistent with much of what we know about sensorimotor functions, emotion and motivation, and more complex cognitive processes. On the other hand, if we wish to embrace a more embodied view of cognition, we could argue that interactions between the brain and body (visceral and somatic processes) are integral to cognition. Therefore, we may not wish to exclude physiological processes outside the brain in representing mental phenomena. For this reason, we recommend either the second or third view as a reasonable starting point for cognitive ontologies.

Like any complex system, cognition comprises multiple subsystems. Most researchers would agree that cognitive subsystems include at least perception, attention, short- and long-term memory, decision-making, language, and emotion (James, 1890; Poldrack et al., 2011). Consequently, any ontology of mental functioning is likely to include terms for at least these sorts of phenomena. However, many finer-grained distinctions can be drawn. Theories of cognition differ in whether and how they draw these distinctions, and in how the subsystems assumed by the theory are thought to interact and give rise to patterns of behavior.

Mental phenomena cannot be observed in the same way that behavior or brain activity can be observed. A scientific study must therefore “operationalize,” or define, a mental process with respect to a particular experimental framework. To this end, researchers have devised a variety of experimental paradigms (Turner and Laird, 2012). Each paradigm includes a set of explicit instructions for subjects to behave in certain ways (e.g., attend, then push a button) in response to different stimuli (e.g., words or faces). Outcome measures include response time and accuracy, as well as patterns of physiological (bodily or brain) activity. For example, Fliessbach et al. (2010) examined how the strength of a memory (operationalized in terms of response accuracy) depended on the way that people learn, or encode, new information. Some participants were asked to perform an orthographic (letter) judgment, while others were asked to perform a semantic (meaning) judgment. Results showed that memory recall was better for the semantic task, providing support for a familiar construct in psychology of memory, known as “depth of processing.” This study illustrates how experimental variables are used to operationally define a mental phenomenon. At the same time, we note that operational definitions rely on particular measurement methods, and these methods may not be sensitive to all aspects of the phenomenon of interest, or may reflect additional processes, e.g., so-called task demands. Therefore, converging evidence from multiple methods is essential for studies of cognition.

**FIGURE 1:**
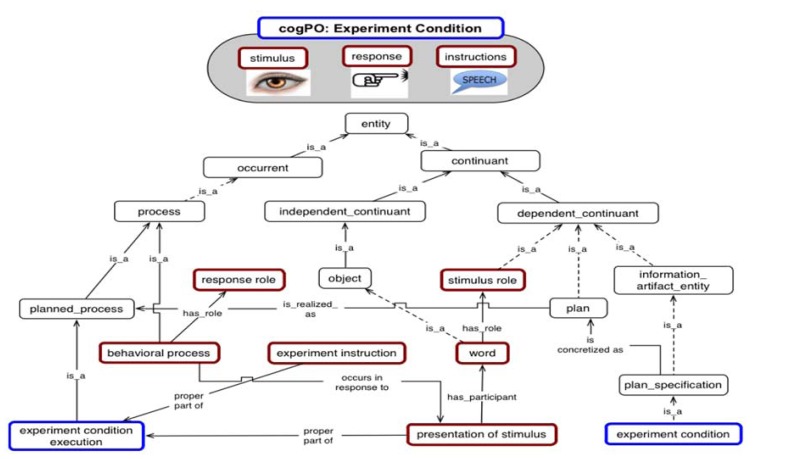
**A partial representation of the Cognitive Paradigm Ontology (CogPO)**.

In this context, the BrainMap database (Laird et al., 2005) and the Cognitive Paradigm ontology (**Figure 1**, Turner and Laird, 2012) have been used to annotate behavioral and brain data in order to test hypotheses about brain-behavior relationships. BrainMap is a large curated repository of cognitive neuroimaging studies that can be used to explore relationships between behavior and patterns of brain activity (mainly from PET and fMRI). It encodes core experiment metadata, including the conditions, tasks, responses and measurement methods. Using this structure, experimental results can be grouped together, based on the objective similarities among the stimuli, task instructions, and measures, regardless of whether the experiment was designed to study a particular subsystem, such as memory, executive function, or attention (Burns and Turner, 2013).

The Cognitive Atlas (Poldrack et al., 2011) represents a complementary effort. Using a wikipedia-style (or “bazaar” approach; Raymond, 1999), the Cognitive Atlas captures a wide range of terminology used in cognitive science, relating operational measures to the types of mental functioning that they are designed to measure. For example, the Cognitive Atlas contains statements such as “‘pattern comparison task’^3^ measures ‘processing capacity”’^4^.

In clinical neuropsychology, behavioral and self-report measures (called “neuropsychological tests”) are widely used for the diagnosis and treatment of patients with psychological and developmental disorders. Neurological assessment rests on the theory that the nature and location of brain damage, either by disease or injury, can be discovered by testing patients’ cognitive functioning. The NeuroPsychological Testing Ontology (NPT^5^) has been used to characterize these tests, which include measures of cognitive abilities, such as short-term vs. long-term memory recall or executive functioning. The Neurological Disease Ontology (ND^6^) classifies entities relevant to neurological diseases, such as Alzheimer’s and multiple sclerosis. Diseases are classified and related to diagnostic criteria and to neuropathology. A key challenge in clinical and cognitive neuroscience is to understand how physiological disorders due to trauma or disease are related to cognitive deficits, such as memory loss or aphasia. We expect that harmonization of the NPT, ND, and similar resources, will support this goal.

## CHALLENGE #2: INTEROPERABILITY OF COGNITIVE ONTOLOGIES

Several cognitive ontologies have been mentioned thus far. In recent years, psychology has embraced methods and insights from the biological sciences, including molecular genetics, evolutionary biology, and physiology (Kandel, 2008). These methods show the brain at multiple orders of spatial resolution, from genes to neural maps to interacting systems. Likewise, medical research has increasingly turned to biological evidence for explanations of mental disorders, such as depression and schizophrenia (Weyandt, 2006). As a result, many bio-ontologies now also include terminology for mental processes. For example, the Gene Ontology (GO), widely used for cross-species annotation of the functions of gene products (Ashburner et al., 2000), includes terms for “cognition,” “learning,” and “memory,” among others. This inter-domain diversity of ontologies including terminology for cognitive entities is accompanied by diversity in definitions and classification.

To coordinate work across domains and promote convergence, the Open Biomedical Ontologies (OBO) Foundry (Smith et al., 2007) provides recommendations on best practices in ontology development, such as re-use of existing terms, community involvement (i.e. being “open”), and standardization of meta-data practices. In our experience, these standards have contributed to the quality and interoperability of neuroscientific and cognitive ontologies. For example, these ontologies have largely adopted BFO as a common upper ontology, and there is extensive reuse of mid-level ontologies, such as OBI, IAO, OGMS, and UBERON, as shown in **Table 1**. This re-use ensures that corresponding terms across ontologies share exactly the same identifier and definition, thus seamlessly harmonizing the resulting data annotations.

**Table 1:** An overview of ontology efforts for cognitive processes and related domains.

Name/RRID	Location	Status	Citation	Scope	Upper	Related
Mental Functioning Ontology (**MF**)/RRID:nlx_157305	http://purl.bioontology.org/ontology/MF	Alpha	Hastings et al. (2012)	Cognitive processes and dispositions	BFO 2.0	OBI/IAO, OGMS
Cognitive Paradigm Ontology (**CogPO**)/RRID:nlx_155537	http://purl.bioontology.org/ontology/COGPO	Release (v1.0)	Turner and Laird (2012)	Cognitive experimental paradigms	BFO 1.1	OBI/IAO, BrainMap
Neural Electromagnetic Ontologies (**NEMO**)/RRID:nif-0000-10899	http://purl.bioontology.org/ontology/NEMO	Release (v3.2)	Frishkoff et al. (2009)	Cognitive and brain processes, EEG and ERP measures	BFO 1.1	OBI/IAO, NIFSTD, CogPO, UO
Neuroscience Information Framework (**NIFSTD**)/RRID:nlx_144512	http://purl.bioontology.org/ontology/NIFSTD	Release (v2.9)	Imam et al. (2012)	Brain anatomy (cross-species)	BFO 1.1	OBI/IAO, UBERON, CogPO
Cognitive Atlas (**COGAT**)/RRID:nif-0000-24591	http://purl.bioontology.org/ontology/COGAT	Release (v1.0)	Poldrack et al. (2011)	Cognitive processes, cognitive experimental paradigms	–	CogPO
Neurological Disease Ontology (**ND**)/RRID:nlx_157304	http://code.google.com/p/neurological-disease-ontology/	Alpha	Jensen et al. (2013)	Neurological disorders, neuropathology	BFO 2.0	OBI, GO, OGMS, CL, ChEBI, PR
Neuro Behavioral Ontology (**NBO**)/RRID:nlx_151745	http://purl.bioontology.org/ontology/NBO	Beta	Gkoutos et al. (2012)	Behavioral processes and phenotypes	–	GO, ChEBI, UBERON
NeuroPsychological Testing Ontology (**NPT**)/RRID:nlx_157303	https://code.google.com/p/neuropsychological-testing-ontology/	Alpha	Cox et al. (2013)	Cognitive assessment, behavioral measures	BFO 2.0	OBI/IAO

*OBI, Ontology of Biological Investigations; IAO, Information Artifact Ontology; OGMS, Ontology for General Medical Science; UO, Units Ontology; UBERON, Uber Anatomy Ontology; CL, Cell Ontology; ChEBI, Chemical Entities of Biological Interest Ontology; PR, Protein Ontology. All ontologies are available via http://www.obofoundry.org/*

A guiding principle of the OBO Foundry is that of ontological realism, which suggests that ontologies should aim “to identify the sorts of entities that exist... according to the best current scientific understanding” (Smith and Ceusters, 2010). Ontological realism is a foundation for BFO and, in our view, has effectively promoted a community-wide focus on representing scientific knowledge. Interestingly, ontological realism might be seen to be challenged by the need to represent certain mental phenomena, such as hallucination, that can reference entities that do not exist. One solution is to distinguish these processes, which can be viewed as non-canonical, from canonical perception, in which perceptual representations are triggered by sensory stimuli. Perception is *relational* in the sense that it brings the subject into contact with an external object in the world (Mulligan and Smith, 1986). Just as the Foundational Model of Anatomy (FMA, Rosse and Mejino, 2003) contains terms for arm and leg, but not for amputation stump or shriveled arm, ontologies that represent canonical mental processes would contain terms for visual and aural perception, but not for synaesthesia or tinnitus. Terms like these would belong in extension ontologies relating to specific types of mental or neurological disease (Ceusters and Smith, 2010). By focusing on the canonical case, perceptual processes (for example) can safely be defined as representing objects and involving beliefs about those objects. A next challenge, then, is to capture the relationships between canonical and non-canonical processes, acknowledging that some core sub-processes in normal perception are similar, or even identical, to those in (e.g.) hallucinating.

## CHALLENGE #3: APPLICATIONS OF COGNITIVE ONTOLOGIES

In addition to data annotation and integration, ontologies can be used for improved search and retrieval of other resources and for reasoning over data within as well as across experiments.

### INFORMATION RETRIEVAL

The Neuroscience Information Framework^7^ (NIF) provides for search and retrieval across a wide range of resources, including publications, databases and other information sources. The search engine is backed by the NIF ontology (NIFSTD), which supports better formalization and integration of existing resources. For example, the ontologies include semantically based mappings between related terms, such as “cognition” and “mental process.” Therefore, an ontology-based search that includes only one of these terms still provides excellent coverage.

### DATA ANNOTATION

Ontologies are increasingly used for data annotation. For example, the behaviors of model organisms are widely used as proxies for the study of mental processes and disorders in humans (e.g. Cryan and Holmes, 2005; Wu and Luo, 2005; Mathur and Guo, 2010). Building on the GO, the NeuroBehaviour Ontology (NBO, Gkoutos et al., 2012) represents behavior and is used for model organism data annotation. Formalization of the relationships between the behavior and assumed homologies in mental functioning represented in ontologies would support better data integration and translation in this domain.

### ONTOLOGY-BASED ANALYSIS

Finally, ontologies can be used to reason across data, e.g., for classification of complex patterns (as supported by NEMO, Bilder et al., 2009; Frishkoff et al., 2009, 2011a,b; Poldrack et al., 2012).

To enable cross-domain applications, Hastings et al. (2012) have proposed a Mental Functioning Ontology (MF) which includes inter-ontology *bridging modules*. A bridging module assigns semantic relationships between terms in different ontologies, such as identity, parthood, or realization. Bridging modules exist independently of each of the source ontologies but enable applications to safely draw on both of the ontologies, harnessing the knowledge-based relational links between them. Our vision is that MF will support harmonization of search and analysis tools across biochemical, biological, and medical data. In doing so, we wish to achieve stronger links between biological research (“bench sciences”), translational research, and medical records. Due to the explicit relationship between disorder categories and lower-level mental processes, we further anticipate that MF might help to further disambiguate data in diagnostic categories for mental disorders that show high levels of co-morbidity (Hastings and Schulz, 2012).

## OUTLOOK: WHAT IS NEEDED FOR THE FUTURE?

In the last few years, multiple ontologies have been created for representation, annotation, and reasoning about cognitive functioning, and for the representation of related behavior and brain data in different experimental contexts. **Table 1** gives an overview of these efforts. At present, there is no one project that unites these efforts, although there is considerable cross-pollination, as reflected in the “Related” column, which shows mid-level ontologies that are shared across projects.

For researchers, mapping between different ontologies can present challenges, as independent ontologies may reflect different assumptions, methodological practices and scientific viewpoints (as reflected, for example, in conflicting definitions). It is inefficient and prone to error for each consumer of the combined research data to re-do the mapping independently, and the difficulty in harmonizing hinders the integration of the knowledge contained in their respective annotations into a broader understanding that spans genetic, molecular, cellular and psychological levels of description. As the underlying science progresses, each of these ontologies will evolve in slightly different ways, rendering any mapping potentially out of date in the future.

Rather than *post hoc* mapping efforts on a project-by-project basis, what is needed is a coordinated international effort towards ontology integration. Ontology integration involves the creation of bridging modules between ontologies that accurately reflect the shared understanding of the semantic relationships between the entities in the different ontologies. This is necessary in order to allow comparison and alignment (and thus maximally effective use) of empirical research data flowing from different sources and methodologies. Since the ontologies and the annotations (data) are separated, ontology-based integration does not change the way researchers in different areas work and in no way forces unwanted theoretical or methodological frameworks onto them. Rather, ontology-based integration flows from the use of a set of common and integrated ontologies to annotate their data. We believe that with some coordinated effort it will be possible to create such common ontologies (in fact, we have already taken some steps in this direction, as described above) in a way that remains neutral as between different methodological approaches and fundamental assumptions.

We believe is the way forward includes (a) continued use of existing ontologies for data annotation by primary data-generating researchers and deposition of those annotated data into shared repositories, alongside (b) ontology-based integration through the creation of shared semantic bridging modules between different ontologies and the increased convergence (as recommended by the OBO Foundry) on re-use of shared components between different ontologies, shared upper and mid-level ontologies, and shared metadata standards.

We are currently building on existing active collaborations among ontology developers, and working towards creating a set of resources that the diverse interdisciplinary community of researchers will be able to stand behind. This will require a large-scale coordinated effort. However, in this era of increasingly data-driven science, as the focus turns to carefully retaining and making data available to drive reproducibility, we believe that there has never been a greater need, and also not a better opportunity.

## CONCLUSION

We have described recent efforts in the development of cognitive ontologies and their use in annotation, integration and analysis of clinical and cognitive neuroscience data. A cross-cutting goal is to identify classes of behavior and brain activity that are related to different types of mental processes. This ambitious aim is consistent with recent calls for large-scale brain-mapping and support for translational neuroscience (e.g., Neuroimaging Informatics Tools and Resources Clearinghouse; Human Brain Project; the Brain Initiative – Devor et al., 2013), projects that are likely to benefit from the development and harmonization of cognitive ontologies. There is a pressing need for coordinated efforts across related disciplines. The NIF plays a key role as an interconnecting hub and primary consumer for many of the ontology efforts described here. There is a real need to provide clear and community-negotiated definitions for core high-level entities within the domain, but for this effort to succeed, wide-scale community participation must be achieved.

## Conflict of Interest Statement

The authors declare that the research was conducted in the absence of any commercial or financial relationships that could be construed as a potential conflict of interest.

## Footnotes

1 For the purposes of this article, the authors agree to use “mental process,” “mental functioning,” and “cognition” synonymously. Distinctions are often made between these, and while there can be good reason to do so in certain cases, clarifying the distinctions here would go beyond our scope.

2 http://bioportal.bioontology.org/search?query=memory&commit=Search

3 http://www.cognitiveatlas.org/task/id/trm_4da86cb034ff6

4 http://www.cognitiveatlas.org/concept/id/trm_4a3fd79d0adf4

5 http://code.google.com/p/neuropsychological-testing-ontology/

6 http://code.google.com/p/neurological-disease-ontology/

7 http://www.neuinfo.org/
